# Determinants of health-promoting behaviors in pregnant women

**DOI:** 10.1590/1806-9282.20231798

**Published:** 2024-07-19

**Authors:** Guven Bektemur, Esra Keles, Leyla Kaya, Kurşad Nuri Baydili

**Affiliations:** 1 University of Health Sciences Turkey, Hamidiye Faculty of Medicine, Department of Public Health – İstanbul, Turkey.; 2 University of Health Sciences Turkey, Zeynep Kamil Women and Children's Disease Training and Research Hospital, Department of Obstetrics and Gynecology – İstanbul, Turkey.; 3 University of Health Sciences Turkey, Hamidiye Faculty of Medicine, Department of Biostatistics – İstanbul, Turkey.

**Keywords:** Health promotion, Pregnancy, Social support, Lifestyle

## Abstract

**OBJECTIVE:**

The aim of the study was to examine the relationship between social support, marital dissatisfaction, psychological factors, and health-promoting behaviors in pregnant women.

**METHODS:**

This cross-sectional study was conducted on 1,265 pregnant women who visited the outpatient clinic of a maternity hospital between May and August 2023. The Health Promotion Lifestyle-II Questionnaire was used to measure the healthy lifestyle behaviors of pregnant women. The mental health status of pregnant women was measured using the Depression Anxiety and Stress Scale-21. The Marital Disaffection Scale was used to assess the level of disaffection toward a spouse. Perceived social support was measured by the Multidimensional Perceived Social Support Scale.

**RESULTS:**

Pregnant women had a mean age of 26.46±5.09 years. Multivariate linear regression analysis revealed that there was a positive association between perceived social support and health-promoting behaviors. It was also found that marital disaffection was negatively associated with health-promoting behaviors (p<0.001).

**CONCLUSION:**

The present study suggests that stress, anxiety, depression, and marital disaffection are negatively associated with health-promoting lifestyle behaviors, while social support is positively associated with the adoption of health practices in pregnant women. Understanding the complex interplay between psychosocial factors and healthy behaviors is crucial to improving healthy behaviors in pregnant women.

## INTRODUCTION

Unhealthy behaviors and lifestyles are responsible for a significant number of deaths globally^1^. During pregnancy, women undergo significant mental and physical changes that can impact their lifestyle and behaviors. Therefore, promoting healthy behaviors during this transformative period is crucial to ensure the well-being of both the mother and the developing fetus^2-4^.

Health-promoting behaviors encompass a range of practices that contribute to favorable health status and pregnancy outcomes. These practices include nutrition, physical activity, health responsibility, stress management, interpersonal relationships, and self-actualization^5^. For instance, there is an increased risk of adverse birth outcomes for pregnant women who are overweight or obese, or who experience greater weight gain during pregnancy. Smoking during pregnancy is related to higher rates of low birth weight and fetal heart rate abnormalities. High levels of stress during pregnancy have also been shown to significantly impact pregnancy outcomes^6^.

Health promotion is a process that enables individuals to have better control over their behaviors. Social, environmental, cultural, and political factors and individual characteristics play a critical role in health promotion. Social support is a major contributor to health promotion, both through behavioral and psychological means. Furthermore, social support may positively impact mental and physical health by reducing stress levels^7^.

Healthcare providers need to be aware of the intricate connection between social support, marital disaffection, psychological factors, and healthy habits in pregnant women in order to promote maternal and child health^8,9^. Effective interventions should focus on removing barriers and strengthening facilitators, with an emphasis on moderating social factors and personal expectations. However, there is a lack of evidence-based interventions in this area due to the varying prevalence rates of psychological factors in different settings. A deeper understanding of the complex interplay between social factors and healthy behaviors is needed. Therefore, the present study aims to explore the association between social support, marital disaffection, psychological factors, and health-promoting behaviors in pregnant women.

## METHODS

This cross-sectional study was conducted on pregnant women who attended the outpatient clinic of a maternity hospital between May and August 2023. The research was conducted under the ethical standards set by the Helsinki Declaration. The Scientific Research Ethics Committee granted approval for the study (approval number: 21/30). All pregnant women gave written informed consent after being fully informed about the study.

Data were collected by a trained midwife under the supervision of the principal investigator. The principal investigator followed the data collection procedure plan. The data were collected using a structured questionnaire administered by a face-to-face interviewer. The data collector received a 3-day training on data collection, sample selection, study tool administration, and data handling procedures. The data collector was familiar with the study's objectives, methods, and ethical aspects.

The inclusion criteria for this study were singleton pregnant women between the ages of 18 and 45 years, who had a gestational age greater than 28 weeks, gave birth to a healthy infant, and were literate. Exclusion criteria were high-risk pregnancy or experienced any stressful life events within the past 6 months. In addition, women with major depression or psychiatric illness, those who delivered babies with anomalies, and those who refused to participate were excluded.

The sociodemographic data form created by the researchers included questions about age, income, family type, body mass index (BMI), education level, and occupation of pregnant women and spouse.

The Health Promotion Lifestyle-II Questionnaire (HPLP-II) was developed by Walker et al.^5^ and later adapted into Turkish by Esin^10^. It consists of 52 items measured on a four-point Likert scale from 1 to 4 (never, 1; sometimes, 2; often, 3; and always, 4). The assessment's total score ranges between 52 and 208 points, with higher scores indicating better health-promoting behaviors. The scale evaluates six dimensions of individual behavior that contribute to a healthy lifestyle: self-efficacy, responsibility for health, interpersonal relationships, stress management, exercise and physical activity, and nutrition. The HPLP-II showed high internal consistency with a Cronbach's alpha value of 0.91.

Depression Anxiety and Stress Scales-21 (DASS-21) was created by Lovibond and Lovibond^11^ and then adapted to Turkish by Yılmaz et al.^12^ to assess participants’ mental health status. The DASS-21 has been empirically validated with various populations from diverse cultures, demonstrating high internal consistency^13-15^. This 21-item scale consists of three dimensions: anxiety, depression, and stress. Each item is scored on a 4-point Likert-type scale. A higher total score is indicative of more severe symptoms of depression, anxiety, and stress. Cronbach's alpha internal consistency ranges between 0.755 and 0.822.

Marital Disaffection Scale (MDS) was developed by Kayser^16^ and adapted to Turkish by Çelik^17^, and was utilized to assess the level of disaffection towards a spouse. It is a 21-item, 4-point Likert-type scale with a single-factor structure. Each item is scored from 1 to 4 (Not at All True, 1; Not Very True, 2; Somewhat True, 3; and Very True, 4). The total score ranges from 21 to 84, with higher scores indicating higher levels of marital disaffection. The scale has high internal consistency, as reflected by the Cronbach's alpha coefficient of 0.89.

Multidimensional Scale of Perceived Social Support (MSPSS) is a psychometric tool designed by Zimet et al.^18^ and adapted to Turkish by Eker et al.^19^ to measure individuals’ perceived social support from three sources: "family, friends, and a significant other." This scale comprises 12 items, rated on a 5-point Likert-type scale from strongly disagree to strongly agree. The internal consistency coefficients reported for the scale are within the range of 0.80 to 0.85, with a Cronbach's alpha coefficient of 0.92.

### Statistical analysis

Statistics were performed with the IBM SPSS 25 software. Qualitative variables were presented as frequencies and percentages, while quantitative variables were presented as means and standard deviations. Independent-sample t-tests were employed for comparisons between variables with two categories and quantitative variables, whereas one-way ANOVA was employed for variables with more than two categories. Pairwise comparisons between categories were done using the Tukey test if a significant difference was detected. To examine the relationship between two quantitative variables, we employed the Pearson correlation. Additionally, we conducted multivariate linear regression to determine the variables influencing the HPLP-II. The significance level was set at p<0.05.

## RESULTS

This study included 1,265 pregnant women who met the eligibility criteria. The pregnant women had a mean age of 26.46+5.09 years. Among the participants, 37.3% had completed secondary school, 84.9% were housewives, and 36.0% had husbands who graduated from secondary school. A great majority of the women (97.5%) had a nuclear family, and 66.6% of the women had a monthly income that was equal to their expenses.

Pregnant women with primary school education had lower HPLP-II scores than those pregnant women with higher levels of education (p<0.001), and those whose spouses graduated from primary school had lower scores compared to those who graduated from university (p=0.017). Pregnant women with income equal to expenses showed higher HPLP-II scores than those with expenses higher than their income (p<0.001). Pregnant women in nuclear households exhibited higher HPLP-II scores than those in extended households (p=0.031) (Table 1).

**Table 1:** The mean Health Promotion Lifestyle-II Questionnaire scores and sociodemographic characteristics.

Variables		HPLP-II Mean±SD	t/F	p-value	Post-hoc comparisons
Educational status					
	Primary school	130.34±26.64	7.332	<0.001*	1<2,3,4
	Secondary school	139.75±27.19			
	High school	138.75±27.12			
	University	144.1±32.23			
Income					
Income is less than expense		133.34±29.27	-5.417	<0.001	2>1
Income is equal to expense		142.46±27.68			
Educational status of spouse					
	Primary school	134±26.53	3.417	0.017*	1<4
	Middle school	139.1±27.66			
	High school	138.43±26.88			
	University	143.32±32.2			
Family type					
	Nuclear family	139.69±28.58	2.157	0.031*	1>2
	Extended family	128.52±24.9			

HPLP-II: Health Promotion Lifestyle-II Questionnaire.

* p<0.05.

The study found no significant correlations between HPLP-II scores and age or pre-pregnancy BMI. However, there was a weak positive correlation between HPLP-II scores and the MSPSS total score and a weak negative correlation between the MDS total score. In addition, a negative correlation was observed between the HPLP-II score and depression, anxiety, and stress scores (all p<0.001) (Table 2).

**Table 2:** Relationship between Health Promotion Lifestyle-II Questionnaire and marital disaffection, multidimensional perceived social support, and Depression Anxiety and Stress Scale-21.

		Total HPLP-II
Age	r	−0.057*
	p	0.044
Pre-pregnancy BMI	r	−0.064*
	p	0.023
MSPSS-total	r	0.309**
	p	<0.001
MDS-total	r	−0.314
	p	<0.001
DASS depression	r	−0.221**
	p	<0.001
DASS anxiety	r	−0.194**
	p	<0.001
DASS stress	r	−0.228**
	p	<0.001

MDS: Marital Disaffection Scale; DASS: Depression Anxiety Stress Scale; MSPSS: Multidimensional Scale of Perceived Social Support; BMI: body mass index; HPLP-II: Health Promotion Lifestyle-II Questionnaire.

* p<0.05.

** p<0.001.

It was found that increases in educational status (β=0.191, p=0.008) and MSPSS scale scores (β=0.196, p<0.001) led to increases in HPLP-II scale scores, while an increase in MDS scores led to a decrease in HPLP-II scale scores (β=−0.195, p<0.001). Age (β=0.191, p=0.570), pre-pregnancy BMI (β=0.191, p=0.336), income (β=0.191, p=0.856), family type (β=0.191, p=0.090), husband's educational status (β=0.191, p=0.079), depression score (β=0.191, p=0.269), anxiety score (β=0.191, p=0.161), and stress score (β=0.191, p=0.181) had no significant relationship with HPLP scores (Table 3).

**Table 3:** Predictors of the pregnant women's Health Promotion Lifestyle-II Questionnaire scores using multivariate linear regression analysis (n=1,265).

	B	Std. Error	St. B	t	p	95%CI
(Constant)	132.289	11.32		11.687	<0.001*	−0.331, −0.182
Age	−0.074	0.131	−0.015	−0.568	0.570	−0.656, −0.224
Pre-pregnancy BMI	−0.216	0.224	−0.026	−0.963	0.336	1.507, −10.014
Educational status	5.761	2.168	0.191	2.657	0.008	−3.016, −3.633
Income	0.309	1.694	0.005	0.182	0.856	−17.585, −1.286
Family type	−8.149	4.809	−0.044	−1.694	0.090	−8.205, −0.45
Educational status of spouse	−3.877	2.206	−0.126	−1.758	0.079	0.39, −0.694
MSPSS-total	0.542	0.077	0.196	7.005	<0.001*	−0.754, −0.423
MDS-total	−0.589	0.085	−0.195	−6.964	<0.001*	−1.109, −0.31
DASS depression	−0.4	0.362	−0.071	−1.106	0.269	−0.192, −1.154
DASS anxiety	0.481	0.343	0.075	1.403	0.161	−1.242, −0.235
DASS stress	−0.503	0.376	−0.085	−1.338	0.181	−0.331, −0.182

MDS: Marital Disaffection Scale; DASS: Depression Anxiety Stress Scale; MSPSS: Multidimensional Scale of Perceived Social Support; BMI: body mass index; HPLP-II: Health Promotion Lifestyle.

* *p* <0.001.

## DISCUSSION

The present study revealed an inverse correlation between stress, anxiety, depression, and marital disaffection with health-promoting lifestyle behaviors and a positive correlation between social support and the adoption of healthy behaviors.

The relationship between social support and health promotion practices in women is complicated and not well understood. The present study found that social support increases health-promoting behaviors. Similarly, Fathnezhad-Kazemi et al.^20^ and Jung and Chun^21^ found that women with higher social support demonstrated better performance in adopting health-promoting lifestyles. However, some studies have failed to demonstrate such an association. It has been reported that support provided by others can sometimes have a negative or ineffective impact, although some studies have reported a positive relationship. Differences in study design and research communities could account for the varying results, highlighting the need for prospective studies that assess pregnant women at each trimester.

Research on the relationship between mental health and health-promoting behaviors has yielded mixed results. While some studies have found a negative relationship, others have found no such relationship. For example, several studies found that there was a significant inverse relationship between pregnancy anxiety and the overall health-promoting behavior score^22,23^. Kemp and Maker^24^, on the contrary, did not find such a relationship between anxiety levels and overall HPLP-II scores. It is possible that the discrepancies in findings between these studies are due to cultural, environmental, and economic differences.

This study demonstrates a negative correlation between depression symptoms and health-promoting behaviors among pregnant women. This aligns with a recent research that highlighted that depression may act as a direct risk factor in compromising healthy practices by reducing self-care during pregnancy^25^. In addition, our research has established a clear relationship between marital disaffection and poor health behaviors. This finding is in agreement with a previous study that also highlighted the negative impact of marital dissatisfaction on healthy pregnancy lifestyles^6^.

### Limitations

Despite the strengths of our study, such as the large sample size, some limitations need to be considered. One of the limitations of this study is the inability to establish cause and effect due to its cross-sectional design. In contrast to previous studies that examined pregnant women regardless of the trimester, our study only focused on only one-time interval during the pregnancy. Therefore, future studies would benefit more from cohort studies in various trimesters on this issue.

## CONCLUSION

The findings of the current study suggest that stress, anxiety, depression, and marital disaffection are negatively associated with health-promoting lifestyle behaviors, while social support is positively associated with the adoption of health practices in pregnant women. It is therefore crucial to identify psychological risk factors during pregnancy and provide appropriate interventions to enhance the lifestyle of pregnant women. The psychological factors identified in the present study can help healthcare providers develop prevention strategies to promote healthy behaviors in pregnant women.

## ETHICAL STATEMENT

Ethical approval for this study was provided by the Hamidiye Scientific Research Ethics Committee (approval number: 21/30). The database management is in accordance with privacy legislation, and the presented study is in accordance with the ethical principle of the Declaration of Helsinki.

## Data Availability

The dataset used and analyzed in the study is available from the corresponding author upon reasonable request.
